# The mechanism of catalysis by type-II NADH:quinone oxidoreductases

**DOI:** 10.1038/srep40165

**Published:** 2017-01-09

**Authors:** James N. Blaza, Hannah R. Bridges, David Aragão, Elyse A. Dunn, Adam Heikal, Gregory M. Cook, Yoshio Nakatani, Judy Hirst

**Affiliations:** 1 MRC Mitochondrial Biology Unit, Wellcome Trust/MRC Building, Hills Road, Cambridge CB2 0XY, UK; 2 Australian Synchrotron, 800 Blackburn Road, Clayton, Victoria VIC3168, Australia; 3 Department of Microbiology and Immunology, University of Otago, Dunedin 9054, New Zealand; 4 Maurice Wilkins Centre for Molecular Biodiscovery, The University of Auckland, Private Bag 92019, Auckland 1042, New Zealand

## Abstract

Type II NADH:quinone oxidoreductase (NDH-2) is central to the respiratory chains of many organisms. It is not present in mammals so may be exploited as an antimicrobial drug target or used as a substitute for dysfunctional respiratory complex I in neuromuscular disorders. NDH-2 is a single-subunit monotopic membrane protein with just a flavin cofactor, yet no consensus exists on its mechanism. Here, we use steady-state and pre-steady-state kinetics combined with mutagenesis and structural studies to determine the mechanism of NDH-2 from *Caldalkalibacillus thermarum*. We show that the two substrate reactions occur independently, at different sites, and regardless of the occupancy of the partner site. We conclude that the reaction pathway is determined stochastically, by the substrate/product concentrations and dissociation constants, and can follow either a ping-pong or ternary mechanism. This mechanistic versatility provides a unified explanation for all extant data and a new foundation for the development of therapeutic strategies.

Type-II NADH:quinone oxidoreductase (NADH dehydrogenase-2, NDH-2) is a membrane-bound dehydrogenase that oxidizes NADH and reduces quinones and is a central feature of the respiratory chain in many diverse species, but not in animals^1^. Unlike in NDH-1 (respiratory complex I, which catalyzes the same reaction in animals and many other species) catalysis by NDH-2 is not used to support the proton motive force that drives ATP synthesis, so its catalysis is highly exergonic and essentially irreversible. NDH-2 is also much smaller and simpler in structure than complex I – it comprises only a single subunit and one flavin redox cofactor^1^,^2^. Consequently, it has been explored as a gene therapy for human complex I disorders, to relieve the accumulation of NADH and decrease reactive oxygen species (ROS) production^3^,^4^,^5^,^6^. In notable pathogenic microorganisms NDH-2 is essential for growth, even in some organisms that contain both complex I and NDH-2 such as *Mycobacterium tuberculosis*^7^. Together with the presence of NDH-1 in mammalian species, this has rendered NDH-2 an attractive drug target^8^,^9^,^10^,^11^.

Three NDH-2 homologues have been characterized structurally, from *Saccharomyces cerevisiae*^12^,^13^, *Caldalkalibacillus thermarum*^14^, and *Staphylococcus aureus*^15^. All of them are homodimers, with each monomer containing two Rossmann folds that form nucleotide-binding sites for a noncovalently bound flavin adenine dinucleotide (FAD) and the NADH substrate. For the *S. cerevisiae* enzyme (known as Ndi1), the two publications on the structure described contradictory substrate-binding sites. Iwata *et al*. generated co-crystals of Ndi1 with either NAD^+^ or ubiquinone-2 bound (but not both) and found their binding sites to be coincident^12^, precluding formation of a ternary complex (in which both substrates are bound together). Alternatively, Feng *et al*. applied NADH and ubiquinone-4 to NDH-2 crystals together and observed separate densities for the nucleotide and the quinone, so they concluded the binding sites are distinct, allowing formation of a ternary complex^13^. In fact, Feng *et al*. observed two quinone-binding sites (one in both NDH-2:ubiquinone-4 and NADH:NDH-2:ubiquinone-4 co-crystals, the other only in the absence of nucleotide), but the second site is probably an artifact of the high (0.5 mM) ubiquinone-4 concentration used.

The catalytic mechanism of NDH-2 is currently unclear. Kinetic analyses of the *S. cerevisiae, Yarrowia lipolytica, Toxoplasma gondii* and *M. tuberculosis* enzymes using Hanes-Woolf, Lineweaver-Burk and Cook-Cleland reciprocal plots^9^,^16^,^17^,^18^,^19^,^20^ concluded that they follow a ping-pong mechanism in which the substrates bind, react and dissociate sequentially, either in the same binding site (a one site ping-pong mechanism) or in discrete binding sites (a two site ping-pong mechanism). In a ping-pong mechanism, the two substrates are never bound together (Fig. 1A). Alternatively, others have observed charge transfer complexes (CTCs, complexes in an intermediate state between NADH/oxidized flavin and NAD^+^/reduced flavin)^15^,^21^ and suggested that the CTC is the species oxidized by quinone^15^ in a step in which both substrates are bound to the enzyme at the same time. In a classical ternary complex mechanism, both substrates are bound together and both products released simultaneously (Fig. 1B), whereas in NDH-2 formation of a stable CTC suggests that NADH oxidation has already occurred to some extent, before the quinone reacts. Consequently, the ternary complex proposed to be formed during NDH-2 catalysis is atypical because the ternary complex has the character of a substrate-product complex (Fig. 1C). The structures of Feng *et al*.^13^ demonstrated that ternary complex formation is possible, but do not define the mechanism because the observed ternary complex may not be necessary, or even catalytically relevant.

Here, we present a comprehensive analysis of the catalytic mechanism of NDH-2 from *C. thermarum* using kinetic, spectroscopic and structural methods on both the wild-type (WT) enzyme and on site-directed mutants in which the substrate-binding sites have been individually ablated. We find that the two substrate reactions are independent of one another, and that the pathway followed is determined by the relationships between the substrate and product concentrations and enzyme dissociation constants. This mechanistic versatility provides a unified explanation for all extant data, and, by characterizing the specific states and substrate-binding sites required for catalysis, provides a foundation for the future design and evaluation of candidate inhibitors.

## Results and Discussion

### Spectra of NDH-2 define the oxidation and nucleotide-binding states

Figure 2 (left) shows a set of spectra for NDH-2 in different redox and nucleotide-binding states. Panel A shows that addition of NADH to the oxidized enzyme both reduces the flavin (most evident at 350 to 500 nm) and forms a charge-transfer complex (CTC, evident above 550 nm with a maximum at 660 nm)^15^,^21^. Because the reduction potential of the flavin (ca. −0.22 V^15^) is considerably higher than that of NADH (−0.32 V) the CTC is probably best described as NAD^+^ bound to reduced flavin. The CTC is stable, suggesting that NAD^+^ does not dissociate from the reduced flavin following hydride transfer, and panel B shows the CTC is also formed by addition of NAD^+^ to the pre-reduced flavin. Therefore, our data suggest that NAD^+^ remains bound following the redox reaction simply because it is tightly bound, not because it is ‘trapped’ by a conformational change controlled by the flavin’s redox state.

In principle, it is possible to measure the dissociation constant for NAD^+^ by combining these titration data with the tight-binding equation, but the values obtained are inaccurate because the enzyme:substrate complex is always present at close to stoichiometric level. However, the data provide an upper limit for *K*_D_ of 2 μM — considerably lower than the values of 11.4 and 20.3 μM determined for *S. aureus* NDH-2 by titrating NADH and NAD^+^ onto the protein and monitoring the fluorescence from two nearby tryptophans^15^. Our attempts to replicate the same experiments here were unsuccessful due to absorption by the nucleotides at the frequencies used for excitation and/or emission (the inner filter effect). For *S. aureus* NDH-2, the tryptophan residues were excited at 280 nm, and their emission measured at 330 nm. Here, the absorption of both NAD^+^ (which has an absorption maximum at 260 nm that extends to 280 nm) and NADH (maximum absorption at 340 nm) precluded identification of any changes due to nucleotide binding. Significant fluorescence changes were not observed from the flavin in either *S. aureus, C. thermarum,* or *Escherichia coli* NDH-2^22^, monitored at 450 and 530 nm (at which wavelengths the nucleotides do not absorb).

Figures 2A and B (right) further show that the (approximately stoichiometric) NADH + oxidized flavin and NAD^+^ + reduced flavin reactions that generate the CTC are fast and essentially complete within 50 ms (in 2 μM NADH the flavin is reduced at ~200 s^−1^). In contrast, Fig. 2C shows that addition of NADPH reduces the flavin – but no CTC is observed unless a very large excess of NADP^+^ is added to the reduced enzyme, and even then it is only present at low level. Furthermore, the NADPH + oxidized flavin reaction requires more than 10 s, so it is three orders of magnitude slower than the NADH reaction (in 2 μM NADPH the flavin is reduced at ~0.3 s^−1^). Steady-state rates for the NADPH:menadione and NADH:menadione oxidoreduction reactions support this comparison (in 30 μM NAD(P)H and 400 μM menadione the rates were ~1 and ~880 s^−1^, for NADPH and NADH respectively). Together, these observations indicate that the affinity of NDH-2 for NADP^+^/NADPH is much lower than for NAD^+^/NADH. The low NADP^+^/NADPH affinity can be explained by the structure of NDH-2 with NAD^+^/NADH bound (discussed later), by unfavorable interactions between the side chain of E198 and the adenine nucleotide phosphate. Indeed, sequence analyses suggests that the majority of NDH-2 variants contain Glu or Asp at this position and so react only with NADH^2^, and the corresponding Glu to Gln mutation in *Agrobacterium tumefaciens* NDH-2 was demonstrated to change its specificity towards NADPH^23^.

### Flavin oxidation by quinone does not depend on nucleotide binding

**Figure 1:**
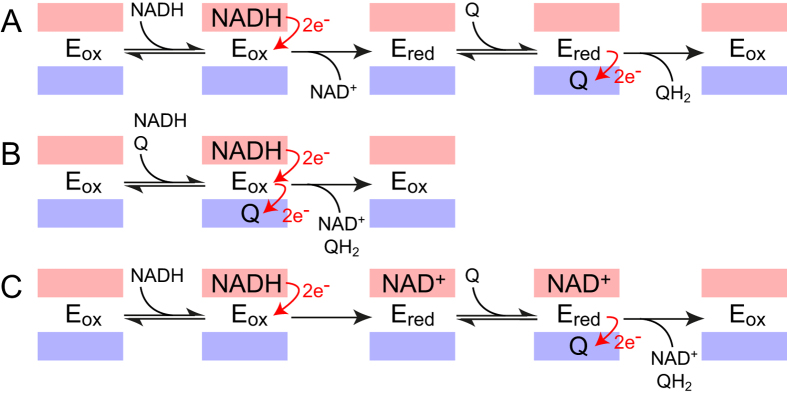
Reaction mechanisms relevant to NDH-2 catalysis. The binding sites for NADH/NAD^+^ and Q/QH_2_ are shown shaded in red and blue, respectively. (**A**) Ping-pong mechanism in which NADH and Q (quinone) react sequentially and are never bound to the enzyme at the same time. (**B**) Classical ternary mechanism in which both substrates bind to form a ternary complex, the reaction occurs, and both products dissociate. (**C**) Atypical ternary mechanism that better reflects proposed mechanisms for NDH-2, in which NADH binds and reacts but NAD^+^ does not dissociate, then Q binds and reacts in a ternary complex with the enzyme-product complex.

**Figure 2:**
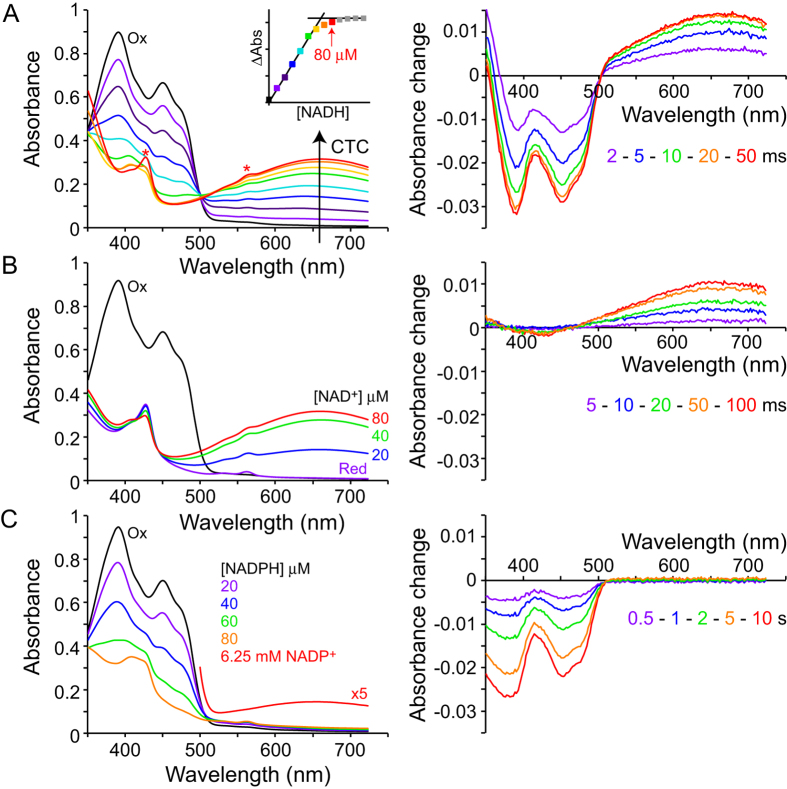
Spectra of NDH-2 in different redox and nucleotide-bound states. (**A**) Left, the spectrum of ~70 μM oxidized NDH-2 is in black (Ox), then subsequent spectra show how the spectrum evolves upon addition of 10 μM aliquots of NADH. Wavelengths below 500 nm show flavin reduction, wavelengths above 500 nm formation of the charge-transfer complex (CTC). The inset shows the CTC absorbance plotted against NADH concentration showing the CTC is formed close to stoichiometrically with NADH. The asterisks mark two signals from a low-level but strongly absorbing contaminant heme protein (which is estimated to be present at less than 2% of the concentration of NDH-2). Right, difference spectra (spectrum at the reported timepoint minus the oxidized spectrum) for the reaction of 4 μM oxidized NDH-2 with 5 μM NADH (concentrations after mixing) showing rapid flavin reduction and CTC formation. (**B**) Left, the spectrum of ~70 μM oxidized NDH-2 is in black (Ox) and that of the dithionite-reduced enzyme in violet (marked Red). Subsequent spectra follow the addition of aliquots of NAD^+^ to generate a final spectrum equivalent to in A. Right, difference spectra (relative to the 1 ms spectrum) for the reaction of 4 μM dithionite-reduced NDH-2 with 4.5 μM NAD^+^ (concentrations after mixing). (**C**) An experiment equivalent to in (**A**) but with NADPH instead of NADH. Left, NADPH reduces the flavin but does not form the CTC; when an extremely high concentration of NADP^+^ is added a very weak CTC signal can be observed (for the 6.25 mM spectrum shown the 80 μM spectrum has been subtracted and the difference spectrum expanded by 5). Right, reduction of 4 μM NDH-2 by 5 μM NADPH (difference spectra relative to the 1 ms spectrum, concentrations after mixing) is much slower than reduction by NADH.

**Figure 3:**
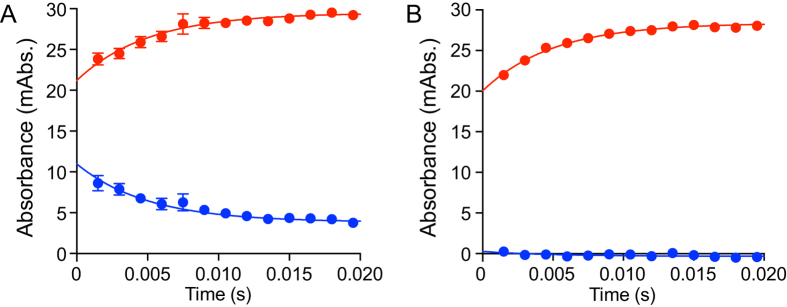
Re-oxidation of the reduced flavin in NDH-2 by menadione in the presence and absence of bound nucleotide. A solution of 8 μM NDH-2 was sub-stoichiometrically pre-reduced by addition of NADH (**A**) or NADPH (**B**) to generate 4 μM reduced enzyme (determined spectroscopically), then loaded into the stopped-flow apparatus. It was reacted at a concentration of 2 μM reduced enzyme with 1.5 μM menadione (concentrations following mixing) with the sub-stoichiometric ratios used to ensure all the reactants are consumed. The data points are average values from three technical replicates with error bars ± SEM (n = 3). The absorbance from the flavin (red, average absorbance from 438 to 464 nm) increases upon oxidation. The absorbance from the CTC (blue, average absorbance from 639 to 684 nm), only evident in the NADH-reduced protein, is lost upon oxidation. Each trace is shown fitted with a first-order rate constant of 200 s^−1^; the best-fit values are 170 (flavin) and 130 (CTC) s^−1^ in A and 220 s^−1^ in (**B**).

Figure 3 shows kinetic traces for oxidation of the reduced flavin by menadione, a short-chain analogue of the physiological menaquinone, either with the nucleotide-binding site occupied by NAD^+^ (Fig. 3A), or unoccupied (Fig. 3B). The NAD^+^-bound enzyme was generated by reduction with a sub-stoichiometric concentration of NADH, and the nucleotide-free enzyme with NADPH. For the NAD^+^-bound enzyme, reoxidation of the flavin by a sub-stoichiometric concentration of menadione is mirrored by loss of the CTC (for the nucleotide-free enzyme the CTC is absent). Importantly, the rate of flavin reoxidation by menadione is essentially identical in both cases (~200 s^−1^ in 1.5 μM menadione), showing that quinone reduction does not depend on the nucleotide-binding site occupancy. Furthermore, the rates of flavin reduction by NADH (Fig. 2) and flavin reoxidation by menadione (Fig. 3) are comparable. These observations demonstrate that NDH-2 catalysis can follow either a ternary-complex mechanism (as with NADH) or a ping-pong mechanism (as with NADPH, Fig. 1A), with the route taken determined simply by the affinity of the oxidized nucleotide for the reduced flavin (and its rate of dissociation). For NADH:menadione oxidoreduction, oxidation of the nucleotide-bound flavin by menadione causes rapid nucleotide dissociation from the oxidized flavin, implying that the NAD^+^ dissociation constant is strongly dependent on flavin oxidation state. Finally, due to the formation of the stable CTC between NAD^+^ and the reduced enzyme, the quinone most likely reacts with the NAD^+^: reduced flavin complex, which is an enzyme-product complex. There is no suggestion that both substrates must bind before reacting together in a common transition state, and so the NADH:menadione oxidoreduction reaction follows an atypical ternary mechanism (Fig. 1C), rather than a classical ternary mechanism (Fig. 1B).

### The NADH- and quinone-binding sites in the NDH-2 structure

**Figure 4:**
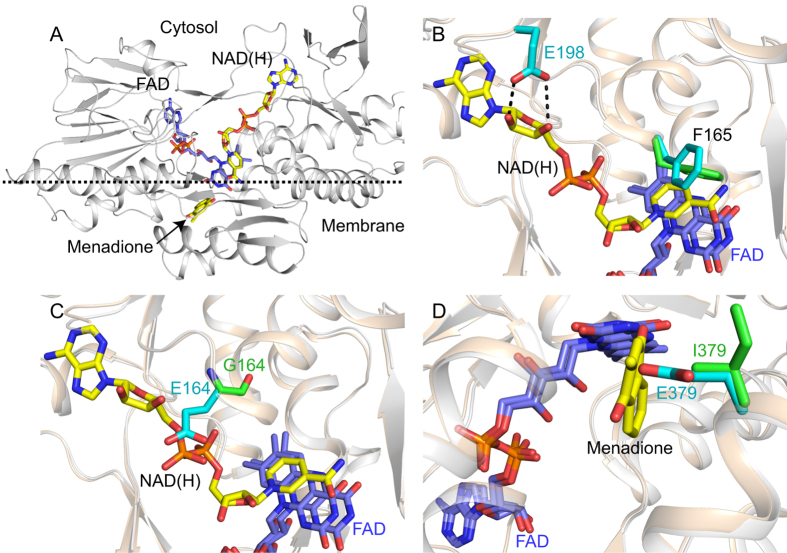
Structures of NDH-2 substrate-binding sites and variants. (**A**) Overview of the structure of NDH-2 showing the location of the membrane interface, the FAD (blue) and two bound substrates (yellow/orange). The structure is for NDH-2 co-crystallized with 1 mM NADH; menadione was modeled into its site as described previously^14^. (**B**) The structures of NDH-2 crystallized in the presence (white) and absence (wheat) of NADH are overlaid. The nicotinamide ring of the nucleotide (yellow) is juxtaposed on the FAD (blue) isoalloxazine ring system at a distance of 3.2 Å (C4′ of the nicotinamide ring to N5 of the FAD). The carboxyl side chain of conserved E198 forms two hydrogen bonds to the adenine ribose and the side chain of F165 changes position (green to cyan) upon nucleotide binding. (**C**) The structures of wild-type NDH-2 (wheat) and the G164E variant (white) are overlaid. NADH has been added for reference only and it clashes with the side chain of E164. (**D**) The structures of wild-type NDH-2 (wheat) and the I379E variant (white) are overlaid. Menadione has been added for reference only and it clashes with the side chain of E379.

The structure of *C. thermarum* NDH-2 has been described, but attempts to determine the structures of its substrate-bound states were previously unsuccessful^14^. Here, we have determined structures of NDH-2 co-crystallized with either 1 mM NADH or 10 mM NAD^+^ to 2.5 and 3.0 Å resolution, respectively. The two nucleotide-bound structures are very similar, so we describe the structure with NADH, which likely represents NAD^+^ bound to the reduced flavin as the product of NADH oxidation. NAD(H) did not alter the crystal packing or unit cell parameters significantly (see Supplementary Table 1) and the RMSD, between the nucleotide-bound and -free structures, was 0.52 Å over 394 Cα atoms. No structure has been determined for *C. thermarum* NDH-2 with a quinone bound, but the high similarity of NDH-2 and Ndi1 readily allowed the bound quinone observed in Ndi1^13^ to be modeled into NDH-2 on the opposite face of the flavin to the nucleotide-binding site (the second, distant site observed in Ndi1 is blocked in NDH-2 by the first amphipathic helix of the membrane-anchoring domain)^14^. Figure 4A shows an overview of the structure of *C. thermarum* NDH-2, showing the FAD and both substrates bound. Clear density for the bound nucleotide was observed in the NADH-NDH-2 structure, with the nicotinamide ring juxtaposed over the flavin in a position consistent with hydride transfer, equivalent to that observed in Ndi1^13^,^24^ (see Fig. 4B and Supplementary Fig. 1). Interestingly, although few changes can be identified between the nucleotide-bound and nucleotide-free structures, the side chain of Phe165 moves away from the *re*-face of the FAD upon nucleotide binding, as a conserved feature of FAD-dependent reductases that use NADH or NADPH^25^, and the side chain of Phe207 shifts to avoid Phe165. The close similarity of the two structures is consistent with our proposal that NAD^+^ is retained at the reduced flavin simply because of its high affinity, not because of a conformational ‘switch’ that must open to allow it to dissociate.

### Mutations that target the NADH- and quinone-binding sites

To test the proposal that the two substrate reactions are independent of each other we mutated a key residue in each substrate-binding site. G164 is positioned next to the adenine-proximal ribose of the bound nucleotide and I379 is positioned at the entry to the quinone-binding pocket. Structures of the two mutants (Fig. 4C,D), at 3.2 and 2.7 Å resolution, respectively (Supplementary Table 1), confirmed that the structural changes are localized and the integrities of the structures are maintained. G164E crystallized in a different space group (P*4*_*1*_*2*_*1*_*2*) to the wild-type (WT) protein, but the glutamate side chain could be observed confidently in only one of two molecules in the asymmetric unit. On the other hand, I379E crystallized in the same space group as the WT and the side chain was observed in three of four molecules in the asymmetric unit. In comparison with WT, G164E and I379E exhibit RMSD values of 0.54 Å (Cα of 395 residues) and 0.45 Å (Cα of 393 residues), respectively, with no substantial additional differences.

### Kinetics of the G164E and I379E variants confirm that NADH oxidation and quinone reduction are independent of each other

**Figure 5:**
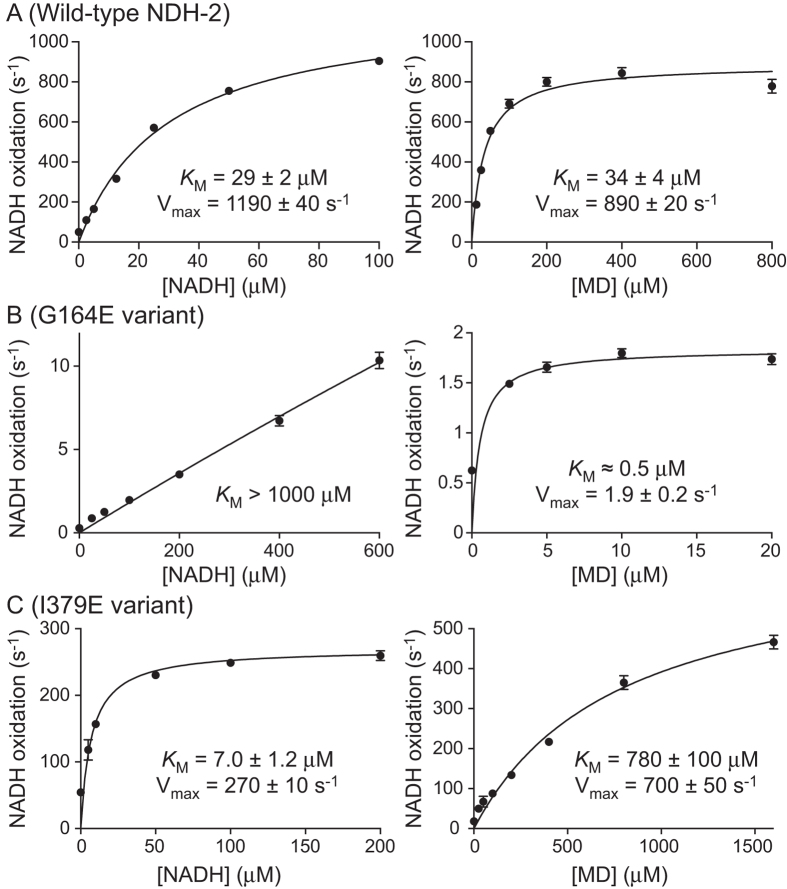
Steady-state kinetic data comparing the wild-type enzyme and NDH-2 variants. Measurements made with varying NADH concentrations used 400 μM menadione, and measurements with varying menadione concentrations used 100 μM NADH. The data were fit to the Michealis-Menten equation. The data points are average values from three technical replicates with error bars ± SEM (n = 3).

Figure 5 shows steady-state kinetic data on WT NDH-2 and its G164E and I379E variants. For WT, the *K*_M_ values are 29 μM for NADH and 34 μM for menadione. For the G164E variant the *K*_M_ for NADH increases drastically to more than 1 mM, consistent with the mutation compromising NADH binding. Furthermore, the observed rates drop drastically (only ~1% of the WT activity is observed in even 600 μM NADH), and, as a consequence, the apparent *K*_M_ value for menadione decreases significantly because the turnover rate is ‘capped’ by the slow rate of NADH oxidation. For the I379E variant, the *K*_M_ for menadione increases significantly, to 780 μM, consistent with the mutation compromising quinone binding. Again, the observed rates are lower, with the maximal rate equivalent to ~60% of the WT rate, and, as expected, the apparent *K*_M_ value for NADH has decreased.

**Figure 6:**
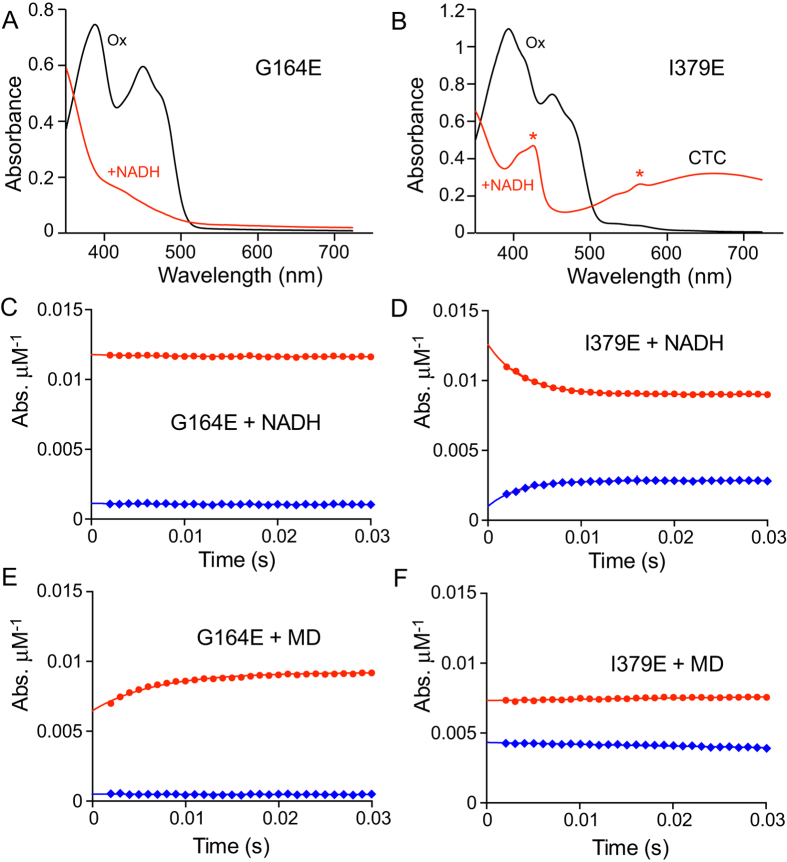
Reactions of the G164E and I379E variants with NADH and menadione. (**A** and **B**) spectra of the oxidized and NADH-reduced G164E and I379E variants. The heme-containing contaminant (marked by asterisks) is clearly visible in I379E (but estimated to be present at less than 2% of the concentration of NDH-2). (**C** and **D**) reactions of oxidized G164E (3.4 μM) and I379E (4 μM) with 2 μM NADH (concentrations following mixing). (**E** and **F**) Solutions of 6.8 μM G164E and 8 μM I379E were sub-stoichiometrically pre-reduced by addition of NADH to generate 3.6 μM reduced G164E and 6 μM reduced I379E (determined spectroscopically), then loaded into the stopped-flow apparatus. They were reacted at concentrations of 1.8 μM reduced G164E and 3 μM reduced I379E with 1.5 μM menadione (concentrations following mixing) with the sub-stoichiometric ratios used to ensure all the reactants are consumed. In (**C**–**F**) the data points are average values from three technical replicates with error bars ± SEM (n = 3), reported in absorbance units per μM enzyme. The absorbance traces are from the flavin (red, average absorbance from 438 to 464 nm, decreases upon reduction) and from the CTC (blue, average absorbance from 639 to 684 nm). G164E does not form a CTC (**A**) and reacts very slowly with NADH (not visible on the experimental timescale, **C**) whereas I379E reacts rapidly with NADH to form a CTC (**B** and **D**). NADH-reduced G164E is oxidized rapidly by menadione (**E**) whereas I379E reacts much more slowly (**F**).

Figure 6A shows that NADH is able to reduce the oxidized G164E variant, but it does not form a CTC. Subsequent additions of NAD^+^ to the reduced protein also failed to produce a CTC. Figure 6C shows that the reaction between G164E and NADH is very slow (~0.2 s^−1^ in 2 μM NADH), similar to the reaction of the WT with NADPH (~0.3 s^−1^ in 2 μM NADPH), consistent with weak nucleotide binding. Conversely, when NADH-reduced G164E was reacted with menadione (Fig. 6E) rapid oxidation of the flavin (~175 s^−1^ in 1.5 μM menadione, matching the ~200 s^−1^ rate for WT) was observed, demonstrating that the quinone-binding site is fully functional, irrespective of the functionality of the NADH-binding site. I379E displays the opposite behavior. When oxidized I379E reacts with NADH, the flavin is reduced and a CTC forms, as in the WT enzyme (Fig. 6B). As in the WT enzyme the reaction with NADH is rapid (Fig. 6D, ~290 s^−1^ in 2 μM NADH), but when the NADH-reduced enzyme is exposed to menadione the reaction is very slow (Fig. 6F, ~3 s^−1^ in 1.5 μM menadione). Therefore, in the I379E variant the menadione reaction is compromised but, irrespective of the functionality of the quinone-binding site, the NADH-binding site functions properly. Our results demonstrate that the decreased steady-state rates observed in Fig. 5 do indeed stem from specific ablation of one of the two active sites, and confirm that the two sites function independently.

### Production of reactive oxygen species

It has been suggested that the tight interaction between nucleotides and the flavin represented by the CTC has evolved to minimize ROS production by the reduced flavin^15^,^21^. Therefore, rates of H_2_O_2_ production were measured in air by monitoring the reduction of Amplex Red to resorufin by horse-radish peroxidase, and confirmed by monitoring NAD(P)H oxidation^26^. The rate of NADH-driven H_2_O_2_ production (0.11 ± 0.01 (n = 4) μmol H_2_O_2_ min^−1^ mg^−1^ or ~0.085 s^−1^) is slower than the NADPH-driven rate (~0.185 s^−1^) measured under the same conditions. Consistent with a small effect of the CTC in minimizing ROS production, little difference in NADH-driven ROS production was observed between the WT and I379E variant (~0.085 *vs*. ~0.088 s^−1^), whereas the rate of NADH-driven ROS production by G164E matches the WT-rate with NADPH (~0.167 *vs*. ~0.185 s^−1^). However, all of these rates are very low in comparison to the rates of catalysis observed with NADH and menadione, questioning whether they are sufficient to drive the evolution of the CTC.

### Evaluation of kinetic data that indicated a ping-pong mechanism for NADH: quinone oxidoreduction

First, NDH-2 was demonstrated to catalyze the transhydrogenation reaction between NADH and thioNAD^+^, an NAD^+^ analogue in which the amide on the nicotinamide ring is replaced with a sulfonamide. Because both NAD(H) and thioNAD(H) must occupy the nucleotide-binding site to exchange a hydride with the flavin, the transhydrogenation reaction was taken as evidence for ping-pong kinetics^20^. Here, we tested the NADH:APAD^+^ reaction, in which APAD^+^ is an NAD^+^ analogue with the amide replaced by an acetyl group and a higher potential (−240 mV at pH 7) than that of NADH (−340 mV)^27^. Michaelis-Menten analyses for the NADH:APAD^+^ reaction showed that *V*_max_ is 2.9 μmol NADH min^−1^ mg protein^−1^ (~500 times slower than for the NADH:menadione reaction), and *K*_M_^APAD+^ is 7.2 μM. Subsequently, titrating APAD^+^ onto the reduced enzyme showed that, like NAD^+^, it binds tightly to form a CTC, although due to the higher potential of APAD^+^ slow flavin oxidation is evident. During transhydrogenation, APAD^+^ must compete with NAD^+^ for the reduced flavin, so the reaction is very slow because NAD^+^ dissociation is very slow. The rate of the NADH:thioNAD^+^ reaction was similarly observed to be ~2000 times slower than the NADH:Q reaction^20^. Thus, the slow transhydrogenation reaction does occur by a ping-pong mechanism, but this does not dictate that the much faster NADH:menadione reaction must do so also. Indeed, menadione reacts at a different site, avoiding the need to ‘wait’ for NAD^+^ dissociation.

**Figure 7:**
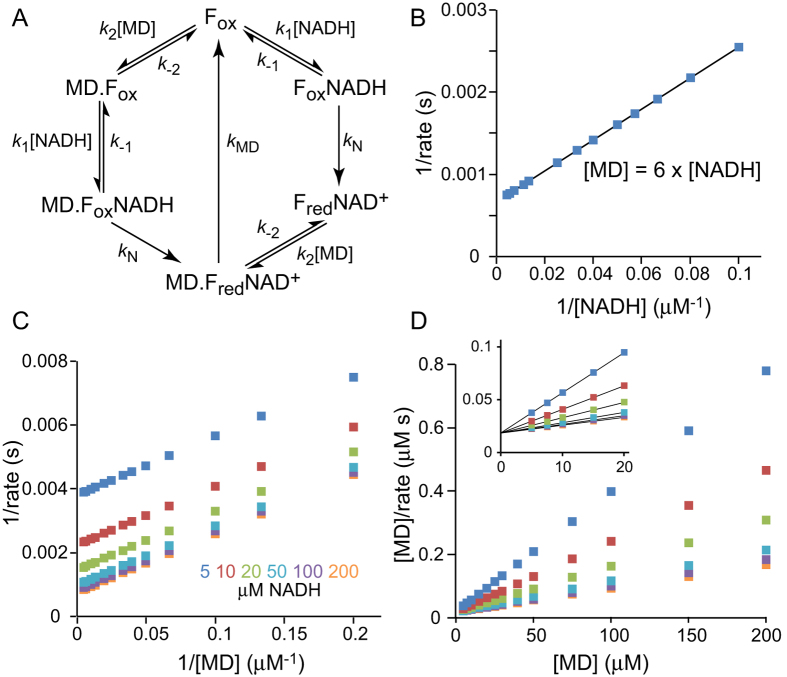
Double-reciprocal analyses for a proposed ternary mechanism. (**A**) A possible ternary mechanism scheme for NDH-2 that includes two branches, depending on whether menadione (MD) or NADH binds first. The constants k_1_ and k_−1_ for NADH binding, k_2_ and k_−2_ for Q binding and k_N_ and k_MD_ for their respective reactions do not depend on the sequence of events. F_ox_ and F_red_ refer to the oxidized and reduced states of the FAD, respectively. (**B**–**D**) Data calculated using the scheme in panel A and a plausible set of parameters determined by trial and error to match the data in Fig. 5: k_1_ = 1.7 × 10^8^ M^−1^ s^−1^; k_−1_ = 5000 s^−1^; k_2_ = 1.1 × 10^8^ M^−1^ s^−1^; k_−2_ = 5000 s^−1^; k_N_ = 3000 s^−1^ and k_MD_ = 3000 s^−1^. (**B**) Cook-Cleland plot with the menadione concentration in a constant ratio of 6:1 with the NADH concentration. (**C**) Lineweaver-Burk plot using the reciprocal menadione concentration for the set of NADH concentrations shown. (**D**) Hanes-Woolf plot for the same data and concentrations shown in panel C. The inset shows the low menadione concentration range and the intersection on the y-axis.

Second, the Cook-Cleland analysis and standard double-reciprocal kinetic analyses were used previously to support proposals for the ping-pong mechanism. Yano and coworkers used Cook-Cleland analyses, in which data are taken at a constant ratio between the two substrates and plotted in double-reciprocal form. The plots are expected to be linear if the reaction follows a ping-pong mechanism, or curved for a ternary mechanism^28^ and the plot for NDH-2 was linear^9^. Two standard forms of double-reciprocal plot analyses have also been used. Parallel lines in Lineweaver-Burk plots, as observed for NDH-2 catalysis^9^,^16^,^18^, are a typical feature of ping-pong reactions^28^, and the same data can also represented by a Hanes-Woolf plot^9^,^17^,^18^,^19^. However, as noted by Cook and Cleland^28^, these analyses only show that the data are compatible with ping-pong kinetics: similar plots can also be produced by reactions with different mechanisms. Figure 7A shows a simple ternary-reaction scheme for NDH-2 catalysis which considers that either NADH or menadione may bind first. Using this scheme the data in Fig. 7B–D were calculated using parameters that reproduce the WT data in Fig. 5: the parameters used are plausible, but many other combinations are also capable of fitting the data in Fig. 5 and more extensive studies would be required to determine their values properly. Figure 7B shows that the ternary mechanism scheme gives rise to a linear Cook-Cleland plot, Fig. 7C shows that it gives parallel lines in a Lineweaver-Burk plot, and Fig. 7D shows the corresponding Hanes-Woolf plot with all the lines intersecting on the y-axis (matching kinetic data presented previously^9^,^17^,^18^,^19^). Therefore, kinetic analyses using these methods are incapable of distinguishing the reaction mechanism for NDH-2 catalysis, and previous conclusions of ping-pong kinetics based on these analyses cannot be relied upon.

### The versatile mechanism of NDH-2 catalysis

The mechanism of NDH-2 is variable: the reaction follows different pathways through the network of possible states depending on the concentrations of reactants and products present, and their different binding constants. During enzyme turnover with NADH and menadione the reaction predominantly follows a ternary-type mechanism in which, following NADH oxidation, NAD^+^ binds tightly to the reduced flavin (forming a CTC) and only dissociates when the flavin is oxidized by menadione (Fig. 1C). In this mechanism, menadione reduction occurs within a ternary product complex. Conversely, during slow enzyme turnover with NADPH and menadione the reaction most likely displays ping-pong kinetics: NADP^+^ does not bind tightly to the reduced flavin so it dissociates rapidly and thus menadione is more likely to react with the nucleotide-free reduced flavin (Fig. 1A). However, the ping-pong mechanism is not enforced, it simply results from the relative rates of the different microscopic reaction steps. Furthermore, our data are consistent with comparable rates for NADH oxidation and menadione reduction, so the identity of the rate-limiting step depends strongly on the relative concentrations of the two substrates. Finally, the NADH- and menaquinone-binding sites operate completely independently of one another, so in all cases the pathway followed for each individual turnover of each independent enzyme molecule is determined stochastically.

### Implications for the development of therapeutic strategies

Our study has revealed how the NADH:quinone oxidoreduction reaction proceeds in two half reactions (NADH oxidation + reduction of the flavin, and re-oxidation of the flavin + reduction of quinone) that occur independently of each other and use spatially distinct substrate-binding sites. Thus, NDH-2 possesses two targetable sites for the development of competitive inhibitors. Furthermore, our new mechanistic information provides a molecular framework by which to understand and validate the modes of action of candidate inhibitors that may relate to either inhibition of NADH or quinone binding. Many compounds, including phenothiazines, quinolinyl pyrimidines, quinolones, nanaomycin A and polymyxin B have been identified as inhibitors of NDH-2 from pathogenic microorganisms^8^,^9^,^19^,^29^,^30^,^31^,^32^, but their modes of action remain unclear. Furthermore, these compounds are not specific to NDH-2, and off-target side-effects have been reported^33^. Defining the catalytic mechanism of NDH-2 is a critical step in establishing a platform for inhibitor validation and may assist in the further application of medicinal chemistry to improve the potency and specificity of candidate inhibitor classes.

## Methods

### Site-directed mutagenesis and enzyme expression and purification

The modified QuickChange method of Liu and Naismith^34^ was adapted to construct the G164E and I379E expression plasmids with pTRCndh2^14^ as a template. *E. coli* C41 (DE3)^35^ cells were transformed with each plasmid, and enzyme expression and purification conducted as described previously^14^. Protein concentrations were measured using the Pierce™ BCA protein assay kit.

### Kinetic and spectroscopic analyses

All kinetic analyses were performed in 50 mM Tris-Cl, 150 mM NaCl, 1% octylglucoside (OG), pH 8.0. The OG provides a hydrophobic volume to accommodate the menadione substrate and maintains the enzyme in an active state. Steady-state kinetic measurements were at 37 °C in a 96-well SpectraMax plate reader and monitored by the absorbance of NADH (340–380 nm, ε = 4.81 mM^−1^ cm^−1^) or APADH (400–450 nm, ε = 3.16 mM^−1^ cm^−1^). Enzyme concentrations used were typically 8 ng mL^−1^ for the wild-type reacting with NADH, but were increased up to 1 μg mL^−1^ for variants/reactions with lower turnover rates. Pre-steady-state measurements were at 23 °C in an SX20 stopped flow spectrometer (Applied Photophysics, dead time ~1 ms) fitted with a photodiode array detector and housed in an anaerobic glovebox. The flavin oxidation state was monitored by the average absorbance over 438 to 464 nm, and the CTC by the average over 639 to 684 nm (taking average values over these wavelength ranges improved the signal:noise ratio). When required, the flavin was pre-reduced using small aliquots of sodium dithionite, NADH or NADPH, then the concentration of reduced enzyme confirmed spectroscopically in control stopped-flow experiments against buffer-only solutions.

### Crystallography

NDH-2 variants were crystallized by hanging-drop vapor diffusion at 18 °C using drops containing 0.5 μL of 30 mg mL^−1^ protein + 1 μL of mother liquor set against 1 mL reservoir solutions. 0.5 μL of seeding stock, prepared using Hampton seeding tubes (100 μL of 0.1 M Bicine/Tris buffer pH 8.5, 10% (w/v) PEG 4000, 25% (v/v) ethylene glycol) were added to each drop to promote crystallization. Typically, small crystals appeared overnight and were harvested on day four. G164E crystallized best in 0.1 M Bicine/Tris buffer (pH 8.5) containing 10% (w/v) PEG 4000, 25% (v/v) ethylene glycol and 30 mM D, L-lysine, and I379E in 55 mM D, L-lysine. The wild-type protein was crystallized with 10 mM NAD^+^ or 1 mM NADH (WT-NAD^+^ and WT-NADH) using the same buffer but without D, L-lysine. Crystals were flash-frozen and stored in liquid nitrogen. Diffraction data were collected at the Australian Synchrotron MX2 beam-line equipped with an ADSC Quantum 315r detector. G164E data were collected using a 20 μm micro-collimator with 0% beam attenuation, 5 s exposures and 1° oscillation angle, and I379E and WT-NAD^+^ data with 30% beam attenuation and 1 s exposures. Five WT-NADH datasets were collected from three crystals with varying attenuation (0–30%) and exposure times (1–2 s) and from a further small crystal using the micro-collimator. Data were processed using XDS^36^. 130° of data from G164E were merged and scaled using Aimless in the CCP4 suite^37^, and similarly 140° for I379E and 160° for WT-NAD^+^. For WT-NADH, 130°, 49°, 160°, 70° and 40° of each dataset were merged. Molecular replacement was performed using Phaser^38^ with a polyalanine WT model (PDB:4NWZ). The structures were refined using PHENIX^39^ with NCS restraints applied, COOT^40^ was used for model building and PyMOL to create the figures. Co-ordinates and structure factors have been deposited in the Protein Data Bank with Accession Numbers 5KMP, 5KMQ, 5KMR and 5KMS.

## Additional Information

**How to cite this article**: Blaza, J. N. *et al*. The mechanism of catalysis by type-II NADH:quinone oxidoreductases. *Sci. Rep.* **7**, 40165; doi: 10.1038/srep40165 (2017).

**Publisher's note:** Springer Nature remains neutral with regard to jurisdictional claims in published maps and institutional affiliations.

## Supplementary Material

Supplementary Information
