# Supplementary material for: The mechanism of catalysis by type-II NADH:quinone oxidoreductases
Source: Sci Rep. 2017 Jan 9;7:40165. doi: 10.1038/srep40165 (PMC5220320; doi:10.1038/srep40165)
Supplement: Supplementary Information [file srep40165-s1.doc]

## The mechanism of catalysis by type-II NADH:quinone oxidoreductases

## James N. Blaza, Hannah R. Bridges, David Aragão, Elyse A. Dunn, Adam Heikal, Gregory M. Cook, Yoshio Nakatani & Judy Hirst

**Supplementary Table 1. Data collection and refinement statistics for NDH-2 crystal structures**

**Supplementary Figure 1**

**
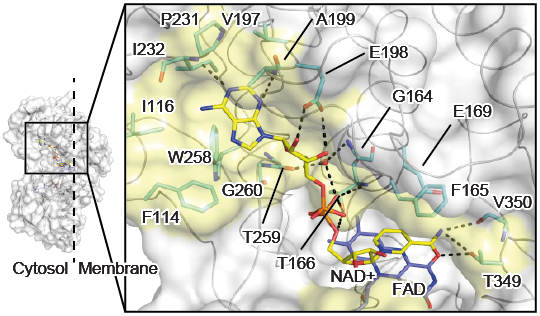
**

**Details of the residues that interact with the bound nucleotide.** The adenine moiety of the bound NAD^+^ sits in the hydrophobic pocket formed by F114, I116, G161, G162, V197, A199, I232, P231 and W258, supported by two hydrogen bonds from the A199 nitrogen to the adenine N3 and from the I232 nitrogen to the adenine N1. The carboxyl side chain of conserved E198 forms two hydrogen bonds to the adenine ribose. The phosphate group adjacent to the nicotinamide nucleotide is held with four hydrogen bonds from the T166 hydroxyl, backbone nitrogen atoms, and a water molecule held by G164 and T259. The nicotinamide ring is positioned between the isoalloxazine ring of the FAD and the side chain of F165, 3.2 Å (C4’ of the nicotinamide ring to N5 of the FAD) from the *re*-face of the isoalloxazine, forming a π-π interaction with it. Further hydrogen bonds are from the T349 hydroxyl and the V350 carbonyl oxygen. Key residues interacting with the nucleotide are shown in cyan, the surface of the NADH binding site is in yellow, the carbons of the NAD^+^ and FAD are in yellow and blue, respectively, and hydrogen bonds are shown with dashed lines.
